# Utilization of systemic inflammatory response syndrome criteria in predicting mortality among geriatric patients with influenza in the emergency department

**DOI:** 10.1186/s12879-019-4288-5

**Published:** 2019-07-19

**Authors:** Henry Chih-Hung Tai, Chien-Chun Yeh, Yen-An Chen, Chien-Chin Hsu, Jiann-Hwa Chen, Wei-Lung Chen, Chien-Cheng Huang, Jui-Yuan Chung

**Affiliations:** 1 Department of Emergency Medicine, Cathay General Hospital, Taipei, Taiwan; 2 Department of Emergency Medicine, Chi-Mei Medical Center, Tainan, Taiwan; 3 Department of Biotechnology, Southern Taiwan University of Science and Technology, Tainan, Taiwan; 4 Fu Jen Catholic University School of Medicine, Taipei, Taiwan; 5 Department of Environmental and Occupational Health, College of Medicine, National Cheng Kung University, Tainan, Taiwan; 6 Department of Senior Services, Southern Taiwan University of Science and Technology, Tainan, Taiwan

**Keywords:** Death, Emergency department, Geriatric, Influenza, Mortality, Prediction, SIRS criteria

## Abstract

**Background:**

Systemic Inflammatory Response Syndrome (SIRS) criteria are often used to evaluate the risk of sepsis and to identify in-hospital mortality among patients with suspected infection. However, utilization of the SIRS criteria in mortality prediction among geriatric patients with influenza in the emergency department (ED) remains unclear. Therefore, we conducted a research to delineate this issue.

**Methods:**

This is a retrospective case–control study including geriatric patients (age ≥ 65 years) with influenza, who presented to the ED of a medical center between January 1, 2010 and December 31, 2015. Vital signs, past history, subtype of influenza, demographic data, and outcomes were collected from all patients and analyzed. We calculated the accuracy for predicting 30-days mortality using the SIRS criteria. We also performed covariate adjustment of the area under the receiver operating characteristic curve (AUROC) via regression modeling.

**Results:**

We recruited a total of 409 geriatric patients in the ED, with mean age 79.5 years and an equal sex ratio. The mean SIRS criteria score was 1.9 ± 1.1. The result of a Hosmer–Lemeshow goodness-of-fit test was 0.34 for SIRS criteria. SIRS criteria score ≥ 3 showed better mortality prediction, with odds ratio (OR) 3.37 (95% confidence interval (CI), 1.05–10.73); SIRS score ≥ 2 showed no statistical significance, with *p* = 0.85 (OR, 1.15; 95% CI, 0.28–4.69). SIRS score ≥ 3 had acceptable 30-days mortality discrimination, with AUROC 0.77 (95% CI, 0.68–0.87) after adjustment. SIRS score ≥ 3 also had a notable negative predictive value of 0.97 (95% CI, 0.94–0.99).

**Conclusion:**

The presence of a higher number of SIRS criteria (≥ 3) showed greater accuracy for predicting mortality among geriatric patients with influenza.

## Background

Influenza is a seasonal disease occurring typically in winter periods. It is highly contagious and spread via airborne or respiratory transmission. During the epidemic season, emergency departments (EDs) may be overwhelmed by patients with influenza. The illness tends to be most severe among elderly people, nursing home residents, infants, young children, and immunocompromised individuals [1]. When illness is associated with complications of pulmonary, cardiovascular, and less frequently, neuromuscular diseases, hospitalization is often required [2]. In the United States, it is estimated that 142,000 hospitalizations are related to influenza infection [3], and approximately 568,000 patient admissions were among elderly adults (age ≥ 65 years) [4]. Furthermore, more than 90% of mortality and complications of influenza occur in elderly patients [3]. Therefore, to most efficiently utilize medical resources, an effective clinical tool is needed to predict the severity of influenza infection among geriatric patients.

The SIRS criteria was initially used as a clinical tool to identify the risk of sepsis and to predict in-hospital mortality. It was first introduced in 1992 by the American College of Chest Physicians (ACCP) and Society of Critical Care Medicine (SCCM) Consensus Conference committee to define sepsis [5]. It is a scoring system, with calculation based on a collection of clinical signs and laboratory investigations that reflect the host inflammatory response, including heart rate > 90 beats per minute, respiratory rate > 20 breaths per minute, body temperature < 36 °C or > 38 °C, White blood cell (WBC) count < 4000/mm^3^ or > 12,000/mm^3^ and band form > 10%. Patients who meet two or more of the SIRS criteria fulfil the definition of SIRS.

Influenza infection may cause a severe inflammatory response, characterized by high fever, muscle soreness [6], and associated symptoms and signs of tachycardia and tachypnea, which are all the variables of SIRS criteria. Although SIRS criteria is no longer the definition of sepsis after the Third International Consensus Definitions for Sepsis and Septic Shock (Sepsis-3), utilization of SIRS criteria in predicting mortality among geriatric patients with influenza has never been investigated. We conducted a keyword search using the terms “death”, “geriatric”, “influenza”, “mortality”, “prediction”, and “SIRS criteria” in PubMed and Google Scholar; however, we found no relevant studies regarding this topic. Therefore, we conducted the present study to delineate the issue.

## Methods

### Study design, setting, and participants

This study was performed at a 800-bed university-affiliated medical center in Taipei, the capital city of Taiwan. Approximately 55,000 patients present to the study ED each year [7], where they are cared for by board-certified emergency physicians. About 33% of these ED patients are elderly adults [8, 9]. In our study, we recruited geriatric patients (age ≥ 65 years) who presented to the ED between January 1, 2010 and December 31, 2015, and who fulfilled the following conditions: (1) tympanic temperature (TM) ≥ 37.2 °C or an increase in baseline TM ≥ 1.3 °C [8, 9], and (2) influenza infection defined as a positive influenza pharyngeal or throat swab test using pharyngeal or throat swab test (de antigen detection) [10].

### Definition of variables and primary outcome

SIRS criteria are defined as heart rate > 90 beats per minute, respiratory rate > 20 breaths per minute, temperature < 36 °C or > 38 °C, WBC count < 4000/mm^3^ or > 12,000/mm^3^ and band form > 10% [5]. The SIRS criteria score will be calculated and obtained while arriving at the ED. Sepsis is defined according to the sepsis-3 campaign. Infected patients with total Sequential Organ Failure Assessment (SOFA) score ≥ 2 points are considered as sepsis. Patients who survived at least 30 days (since arriving at the ED) were considered “survivors” in this analysis [11, 12]. Telephone follow-up was used to ascertain 30-day survival if the patient was discharged from the hospital in less than 30 days.

### Data collection and assignment to case and control groups

A retrospective chart review method was used to obtain data of geriatric patients in the ED who fulfilled the criteria of influenza infection. Patients’ vital signs, demographic characteristics, influenza subtype, laboratory data, past medical history, admission, and 30-day mortality data were collected by an emergency physician. Finally, 479 geriatric ED patients met the criteria of influenza infection. After excluding 70 patients who were lost to follow up, had insufficient data, or transferred patients who had been treated at other hospitals, a total 409 patients were finally included. The included patients were categorized into either the survival and the mortality group, based on their 30-day outcome. All variables were compared between the two groups.

### Ethical statement

This study was approved by the Institutional Review Board of Cathay General Hospital and conducted according to the Declaration of Helsinki. Because this was an observational study, the need for informed consent from patients was waived.

### Statistical analysis

Statistical analysis was performed using SPSS 23.0 for Mac (SPSS Inc., Chicago, IL, USA). The statistical power of this study size (409 patients) was adequate for 0.80, calculated via G-power 3.0. Continuous data are presented as means ± standard deviation (SD). We used an independent samples *t*-test, or the Mann–Whitney–Wilcoxon test for continuous variables in the univariate analyses. Pearson’s chi-square test or Fisher’s exact test was used for categorical variables. We dichotomized SIRS criteria to less than 1 and 1 or more; less than 2 and 2 or more; less than 3 and 3 or more. Logistic regression was then performed to evaluate 30-days mortality prediction among each dichotomized SIRS criteria group (*p* value < 0.05). The area under the receiver operating characteristic curve (AUROC) was used to evaluate mortality discrimination ability. AUROCs were further adjusted for comorbidities that affect mortality (*p* value < 0.1) in regression modeling. The Hosmer–Lemeshow goodness-of-fit test was performed to evaluate the reliability of the scoring system. Sensitivity, specificity, positive predictive value (PPV), and negative predictive value (NPV) were also analyzed.

## Results

A total 409 patients were included in this study. The 30-day mortality rate was 4.9% (20/409) (Table 1). The percentages of the two sexes were equal, and mean patient age ± SD was 79.5 ± 8.3 years. The mean ± SD of systolic blood pressure, heart rate, body temperature, respiratory rate, Glasgow coma scale, and WBC count were 146.1 ± 30.5 mmHg, 98.8 ± 20.5 beats/min, 38.18 ± 0.93 °C, 21.3 ± 4.2 per minute, 13.9 ± 2.3, and 10,590.0 ± 5820.0, respectively. Higher prevalence of cancer was noted in the mortality patient group whereas there was a lower prevalence of chronic obstructive pulmonary disease among patients in the survival group.

**Table 1:** Characteristics of geriatric patients with influenza in the emergency department

Characteristics	Total patients (*n* = 409)	Mortality (*n* = 20)	Survival (*n* = 389)	*p* Value
Male sex	205 (50.1)	13 (65.0)	192 (49.3)	0.17
Age, years	79. 5 ± 8.3	81.2 ± 8.5	79.5 ± 6.4	0.36
Age subgroup				
Young elderly (65–74 yr)	125 (30.6)	4 (20.0)	121 (31.1)	0.31
Moderately elderly (75–84 yr)	174 (42.5)	10 (50.0)	164 (42.1)	0.51
Old elderly (≥85 yr)	110 (26.9)	6 (30.0)	104 (26.8)	0.76
Vital signs				
Respiratory rate (per minute)	21.3 ± 4.2	23.4 ± 7.5	21.1 ± 3.9	0.02
Glasgow’s coma scale	13.9 ± 2.3	12.0 ± 4.0	14.1 ± 2.2	0.03
SBP (mmHg)	146.1 ± 30.5	135.1 ± 31.2	146.7 ± 30.5	0.28
Body temperature (°Celsius)	38.18 ± 0.93	38.19 ± 0.92	38.17 ± 0.93	0.38
Heart rate (beats/min)	98.8 ± 20.5	103.0 ± 21.6	98.6 ± 20.5	0.55
Past history				
Cancer	61 (14.9)	7 (35)	54 (13.9)	0.02
COPD	111 (27.1)	0 (0)	111 (28.5)	0.03
Hypertension	263 (64.3)	17 (85.0)	246 (63.2)	0.06
Coronary artery disease	103 (25.1)	10 (50.0)	93 (23.9)	0.09
Stroke	65 (15.9)	4 (20)	61 (15.7)	0.63
Diabetes	163 (39.8)	7 (35.0)	156 (40.1)	0.66
Laboratory data				
WBC (cells/mm^3^)	10,590.0 ± 5820.0	14530.0 ± 6.2	10380.0 ± 5.8	< 0.01
Bandemia (band form > 10%)	43 (10.2)	7 (35)	36 (9.2)	< 0.01
CRP (mg/dL)	8.2 ± 10.1	11.7 ± 9.2	8.03 ± 10.2	0.09
Platelet (10^3^/mm^3^)	186.2 ± 158.8	198.9 ± 140.3	185.6 ± 159.9	0.68
SIRS	1.91 ± 1.1	2.5 ± 1.1	1.9 ± 1.1	0.03
SIRS ≥2	258 (63.1)	16 (80.0)	242 (62.2)	0.11
SIRS ≥3	126 (30.8)	12 (60.0)	114 (29.3)	< 0.01
Influenza subtypes				
Influenza A	278 (68.0)	12 (60)	266 (68.3)	0.45
Influenza B	120 (29.3)	5 (25)	115 (29.5)	0.67
Influenza A + B	11 (2.7)	3 (15)	8 (2.1)	< 0.01
Influenza vaccination	8 (1.9)	1 (5)	7 (1.8)	0.55
DNR	4 (0.98)	2 (10)	2 (0.5)	0.08
Admission rate^†^	343 (83.9)	20 (100)	323 (83.0)	0.05

Data were presented as % or Mean ± SD. *ED* Emergency Department, *SD* standard deviation, *SBP* systolic blood pressure, *COPD* chronic obstructive pulmonary disease, *WBC* white blood cell count, *CRP* C-reactive protein, *DNR* don not rescue, *SIRS* Systemic Inflammatory Response Syndrome

^†^Admission to general ward or intensive care unit

Laboratory data analysis showed that the mortality group tended to have a higher band form percentage and WBC count, and more coinfection (i.e., influenza A and B) than the survival group. Antiviral drugs like oseltamivir or zanamivir are prescribed immediately, once influenza infection is diagnosed. Among the 20 patients in the mortality group, sepsis accounted for 70% of deaths (14 patients), respiratory failure for 15% (3 patients), and cardiovascular events accounted for 15% of deaths (2 patients with acute myocardial infarction and 1 patient with myocarditis). There was no statistical difference with respect to a “do not resuscitate” order between the mortality and survival groups.

**Fig. 1:**
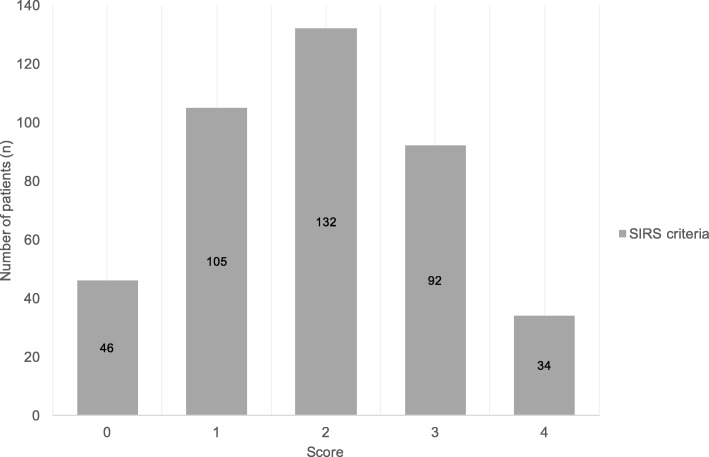
Distribution of patients by Systemic Inflammatory Response Syndrome (SIRS) criteria among geriatric patients with influenza infection

**Fig. 2:**
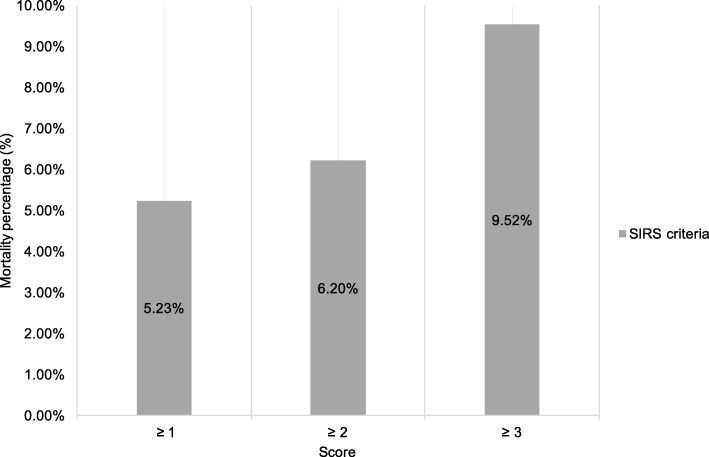
Mortality by Systemic Inflammatory Response Syndrome (SIRS) criteria ≥1, ≥ 2 and ≥ 3 among geriatric patients with influenza infection

The score distribution in geriatric patients with influenza infection, according to the number of SIRS criteria present, showed that 105 patients (25.7%) met 1 criteria, 132 patients (32.3%) met 2, and 92 patients met 3 criteria (Fig. 1). The mortality rate of patients scoring SIRS criteria ≥3 was 9.5, and 6.2% for scoring SIRS criteria ≥2 (Fig. 2). SIRS criteria score ≥ 3 showed better prediction of mortality, with OR 3.37 (95% confidence interval (CI), 1.05–10.73) (Table 2); on the contrary, SIRS score ≥ 2 showed no statistical significance, with *p* value 0.85 (OR, 1.15; 95% CI, 0.28–4.69) (Table 2). The Hosmer-Lemeshow goodness-of-fit was 0.34 for SIRS criteria score ≥ 3.

**Table 2:** Odds ratio, adjusted AUROC and performance of SIRS criteria ≥3 in predicting mortality in geriatric patients with influenza infection

SIRS Criteria	Odds ratio	AUROC	Sensitivity	Specificity	PPV	NPV
	(95% CI)	(95% CI)	(95% CI)	(95% CI)	(95% CI)	(95% CI)
SIRS ≥3	3.37	0.77	0.60	0.70	0.09	0.97
	(1.05–10.73)	(0.68–0.87)	(0.36–0.80)	(0.66–0.75)	(0.05–0.16)	(0.94–0.99)
Hosmer and lemeshow goodness of fit			0.34			

*SIRS* Systemic Inflammatory Response Syndrome, *CI* confidence interval, *AUROC* area under the curve, *AUROC* adjusted by coronary artery disease and cancer, *PPV* positive predictive value, *NPV* negative predictive value

The AUROC, adjusted for coronary artery disease (CAD; *p* = 0.09) and cancer (*p* = 0.02), in predicting mortality showed that SIRS score ≥ 3 (0.77; 95% CI, 0.68–0.87) had acceptable discrimination ability (Table 2). The performance of SIRS score ≥ 3 in predicting mortality among geriatric patients with influenza infection showed good specificity of 0.7 (95% CI, 0.66–0.75) and NPV of 0.97 (95% CI, 0.94–0.99) (Table 2).

## Discussion

Although the new Sepsis-3 guideline no longer uses host inflammatory response syndrome criteria in the identification of sepsis and has eliminated the term severe sepsis [13], SIRS criteria are still useful in predicting the risk of organ dysfunction and mortality in patients with sepsis [13]. There are several articles discussing and debating the usefulness of SIRS criteria in prognosis prediction among adults in the ED and intensive care unit (ICU). Williams and colleagues carried out a large prospective database study at a tertiary Australian medical center, including 8,871 ED patients and aiming to determine the prognostic impact of SIRS criteria. In their research, those authors discovered that SIRS criteria were associated with an increased risk of organ dysfunction (relative risk 3.5) and mortality in patients without organ dysfunction (OR 3.2) [14]. Furthermore, a study in Greece analyzing 3346 patients with infection outside of the ICU and 1,058 patients with infection in the ICU showed that the quick sepsis-related organ failure (qSOFA) score has poorer sensitivity for early sepsis risk assessment than the SIRS criteria [15].

In the present study, we discovered that SIRS criteria score ≥ 3 was a better predictor of mortality among geriatric patients with influenza infection than SIRS score ≥ 2. Similar results were seen in some previous studies among patients with sepsis. A retrospective study of 680 hospitalized patients with bacteremia showed a similar result in that the prognostic sensitivity of SIRS score ≥ 2 was lower than that of SIRS ≥3 in elderly patients aged ≥ 85 years [16]. Another ICU-based study in Greece also indicated that SIRS score ≥ 3 was a better mortality predictor than SIRS score ≥ 2 [15].

A possible reason for our finding is that geriatric patients with infection usually present with ambiguous initial symptoms due to a decreased physiological response [17, 18]. Therefore, a family member or caregiver could easily overlook the patient’s infection status, with a resulting delay in seeking medical attention until disease progression when the illness has become severe. In the present study, the mean duration from initial symptoms to time of ED arrival in geriatric patients with influenza was 3.56 days. Apparent physiological response was seen under this delayed ED arrival circumstances, with mean heart rate > 90 beats per minute, mean body temperature > 38.0 °C, mean respiratory rate > 20 per minute, and mean WBC count > 10,000 mm^3^ (Table 1).

We found that the sensitivity and specificity of SIRS score ≥ 3 were < 80%; however, SIRS ≥3 had a notable NPV of 97%, which may be useful in ruling out poor prognosis and mortality among geriatric patients with influenza [19]. Comorbidities such as CAD and cancer may affect mortality in geriatric patients with influenza infection [20]. Infection with influenza may aggravate underlying cardiac disease, deteriorate heart function, and increase the chance of myocardial infarction in patients with a past history of CAD [21]. Patients with a history of cancer may have undergone chemotherapy or radiation therapy, which may compromise the entire immune system, resulting in immunocompromised status and an increased risk of sepsis [22]. The AUROC was therefore adjusted for these comorbidities.

To our knowledge, this is the first study to report the utility of SIRS criteria in the prediction of mortality among geriatric patients with influenza. There were also some limitations to this study. First, as the study was conducted at a tertiary medical center, influenza disease severity may be more intense among our patient population. Second, some detailed patient information and data may be missing owing due to the study design, which was a retrospective chart review. Further external validation is needed to proof the findings of this study. Third, the diagnosis of influenza should be further confirmed using other advanced examinations, such as reverse transcription polymerase chain reaction, immunofluorescence assay, or viral culture, as the influenza swab test may yield false positive or false negative results. The PPV and NPV for influenza A were 85.7 and 89.8%, and the PPV and NPV for influenza B were 66.7 and 93.9%, respectively [23]. The method used in this study has the advantage of speed and simplicity, for research purposes. Fourth, patient selection according to a positive influenza test rather than a clinical diagnosis may result in underestimation of the actual number of geriatric patients with influenza in the 5-year study period. Patients who were clinically diagnosed with influenza but had a false negative influenza swab test result may have been overlooked and excluded from the study. The influenza swab test has a modest sensitivity of 58–67% and a high specificity of 98% [24]. Fifth, subtypes of influenza virus were not specified, as different strains of influenza virus may result in different fatality rate [26]. Furthermore, specific factors that affect mortality in different strains of influenza virus should be evaluate too [27].

## Conclusion

The presence of a higher score of SIRS criteria (≥ 3) showed greater accuracy than SIRS ≥2 for predicting mortality among geriatric patients with influenza. The high NPV of SIRS criteria ≥3 makes it a useful tool to rule out poor prognosis and mortality in this patient population. Further research is needed to validate the findings of this study.

## Data Availability

The datasets supporting the conclusions of this article are available from the corresponding author on reasonable request.

## Abbreviations

ACCP: American College of Chest Physicians
AUROC: Area under the receiver operating characteristic curve
CAD: Coronary artery disease
CI: Confidence Interval
COPD: Chronic obstructive pulmonary disease
DNR: Do not rescue
ED: Emergency department
ICU: Intensive care units
NPV: Negative predictive value
OR: Odds ratio
qSOFA: Quick sepsis-related organ failure
SCCM: Society of Critical Care Medicine
SD: Standard deviation
SIRS: Systemic Inflammatory Response Syndrome
Third International Consensus Definitions for Sepsis and Septic Shock: Sepsis-3
TM: Tympanic temperature
WBC: White blood cell
